# New Anæsthetics—Theory of Their Action

**Published:** 1861

**Authors:** Charles T. Jackson


### NEW ANÆSTHETICS—THEORY OF THEIR ACTION.

By CHARLES T. JACKSON, M.D.

Substitutes for ether, as anæsthetic agents, are frequently proposed, and some of them have been practically introduced, with some success. None, however, surpass ether in the two most important qualities of efficiency and safety, and there can be no doubt that with respect to safety, ether is far preferable to any and all anæsthetics thus far discovered.

It may prove interesting to the profession, to inquire into the mode of action of the class of bodies known to produce anæsthesia, when inhaled. This subject has been very carefully studied by me from the outset of anæsthetic practice, and with a view to the discovery of some general law.

The first impression among physicians was that the anæsthetic state was merely one of temporary intoxication.

Secondly, the theory of high excitement, followed by corresponding collapse, accounted for the phenomena. Dr. Jno. C. Warren took, at one time, this view of the matter, and spoke of etherization as "devouring the sensibility by high stimulation, and hence a corresponding nervous depression," which was the anæsthetic state. Others have supposed that etherization produced a partial asphyxia; hence the expression early made use of by some of our surgeons, that there was "little difference between hanging, drowning and etherizing."

It has also been alleged that ether absorbed into the sanguineous circulation affects directly the nervous filaments, either at their origin in the medulla spinalis, or in their distributed extremities, or in both these parts. In support of this allegation, it was cited that the direct application of ether to an exposed nerve *destroyed* its sensibility. By the italization of that word, I call attention to the difference between the anæsthetic state of temporary suspension of sensibility, and the destruction of it; for the nerve acted upon directly by ether, does not recover its powers, but is permanently paralyzed.

Another state of the circulation has been observed, which it is hoped would give some clue to the action of anæsthetic agents, namely, that of slackening, and even temporarily wholly suspending the circulation of blood in the capillary or extreme vessels. It was supposed that by thus cutting off a supply of circulating blood stimulus to the sentient extremities of the nerves, sensation was temporarily suspended. The commencement of insensibility in the remote extremities, the feet, legs and hands, seems to indicate that sensation was suspended at those points first.

The French physiologists, Flourens and Longet, are of opinion that the effects of anæsthetics commence, and primarily act on the great nervous centers, the medulla spinalis and medulla oblongata; and that if the full effect reaches the bulb of the medulla, death will take place from total suspension of all the vitat functions of the body.

A more chemical explanation of the action of anæsthetics is that they all abstract oxygen from the blood, and hence reduce its peculiar stimulating powers on the nerves, and that some of them leave poisonous products, while those left by others are innocuous. Thus, as we have formerly stated, chloroform or the ter-chloride of formyl abstracts three equivalents of oxygen from the blood, but, at the same time, unfortunately it deposits, in exchange for the oxygen, three equivalents of chlorine.

Bi-sulphide of carbon, one of the most terrible anæsthetics ever proposed, the dangerous effects of which I have experienced, and have warned the public about seasonably, acts as a powerful de oxidizer of the blood, both the carbon and the sulphur abstracting oxygen, the first producing carbonic oxide or carbonic acid, while the latter forms sulphurous acid. Carbonic oxide and sulphurous acid are poisons, as is also chlorine, before mentioned.

All the acidiferous ethers when decomposed, as they are, in the organs of respiration and circulation, leave their acids in combination with the blood; hence, nitrous and nitric ether are known to destroy life, and hydro-chloric ether and chloride of hydro-carbon undoubtedly act in the same way, and injure the quality of the blood. Acetic ether is not objectionable, since an organic acid is easily decomposed in the processes of respiration, and is removed in the form of carbonic acid and vapor of water, usual products of normal respiration.

Sulphuric ether, as it is improperly called, since its does not contain any sulphuric acid, is a pure hydro-carbon, with one equivalent of oxygen=$C_4$ $H_5$ O. When decomposed by the action of the blood, it may be converted into aldehyde, acetic acid, and lastly into carbonic acid and water, no fixed product remaining in the blood, but all able to be removed by respiratory action. The odor of the breath of a patient who has been etherized, shows that there are exhaled the oxidized products of the ether, and it is known by analysis that a much larger proportion of carbonic acid is exhaled from the lungs during the etherized state, than in the normal condition of the system.

Without finally adopting any theory of the chemical and physiological action of anæsthetics generally, we may, perhaps, be allowed to call attention to a general law, namely, that all very volatile hydro-carbons act as anæsthetics like the others. Thus, it has long been known that benzine, benzole, oil of turpentine, naptha, when inhaled, will all produce the anæsthetic state. The highly volatile oils of coal tar, likewise, possess anæsthetic properties, and one which has recently been tested in surgical practice, known as keroselene, a highly volatile naphtha, seems to be the least offensive of them. It is evidently a very pure hydro-carbon, analogous to highly rectified naptha, and does not contain any oxygen, as is proved by its property of preserving potassium from oxidation, when it is immersed in it. The first samples of keroselene which I tested, two years ago, proved quite irritating to the organs of respiration, but I have learned recently that a purer and more volatile product has been made at Mr. Downer's works, though I have had no opportunity of testing it practically.

It is obvious that there are two or more volatile oils in the keroselene of commerce, and they are separable by graduated distillation. Some care, therefore, is requisite in the preparation of an uniform product; one which may be properly the subject of experiments by inhalation. I would observe that no analysis has yet been made of the keroselene oils.—*Boston Medical and Surgical Journal.*

